# Words of Consolation

**Published:** 1886-10-02

**Authors:** 


### Words of Consolation.

Communications for this column should be addressed, "Chaplain," care of the Editor of The Hospital.

I.

It is intended that a space in this periodical shall be devoted each week to the religious aspects of suffering and sickness. Considering how much we are indebted to Christianity, and therefore to its Founder, for the erection and maintenance of Hospitals, how much of the care, kindness, and self-sacrifice exerted for the benefit of the sick and dying within Hospital walls is owing to the direct influence of Christian education, we should be wanting indeed if we neglected to draw the constant attention of our readers to the manifold and gracious purposes of Almighty God in this wonderful development of His Gospel of goodwill towards men. If the age of miraculous healing is past, it is still true as ever that the Son of Man is present in the fulness of the Spirit, working through the members of His Church, and so healing our sicknesses and carrying our infirmities. Though not put forth in the same way, the same Power is present, and manifested, in the numerous and ever-increasing discoveries of medical science, whereby the yoke of suffering is made easier and the burden of pain more tolerable.

In dealing with the spiritual aspects of suffering and disease, while endeavouring to address some little consolation to the suffering and sick, we shall hope at the same time to meet the wants of physicians, nurses, and attendants. We shall endeavour to speak to each and all of their high calling in Christ Jesus. We shall hope to remind both sufferers and their attendants that endurance, or work, in a hospital, as the case may be, can only be called blessed, only succeed, in so far as they each become a bearer of the Cross of Christ. Many a sick person suffers, but not as a Cross-bearer. Many an attendant ministers, perhaps diligently, but not as a disciple of the Master-healer.

Nearly all hospitals have their chaplains, whose duty it is, of course, to minister to the spiritual wants of the inmates. But the chaplain has often other duties to perform which prevent him from giving so much attention to separate cases as he would. Perhaps he is a parish priest, with many sick folk to attend besides those in hospital. We trust not to interfere with, but rather to supplement his labours. Again, hospitals have their libraries, more or less furnished with books suitable for sick people; but we believe there are many patients who, finding books, or chapters in books, too long or wearisome, may derive comfort from shorter passages such as we hope to furnish in this periodical. Books are better appreciated by the convalescent: the really sick, if they are able to read at all, or to listen to reading, require only a few words at a time. But these must be words calculated to lighten the dark hours of the weary, to cheer the despondent, to encourage the fearful, and to inspire with patience any who are suffering acutely from pain. Such words will find a place in this column, and will be gladly welcomed by us.

